# Extraordinary sensitivity enhancement by metasurfaces in terahertz detection of antibiotics

**DOI:** 10.1038/srep08671

**Published:** 2015-03-02

**Authors:** Lijuan Xie, Weilu Gao, Jie Shu, Yibin Ying, Junichiro Kono

**Affiliations:** 1 College of Biosystems Engineering and Food Science, Zhejiang University, 866 Yuhangtang Road, Hangzhou 310058, P. R. China; 2 Department of Electrical and Computer Engineering, Rice University, Houston, TX 77005, USA; 3 Department of Physics and Astronomy, Rice University, Houston, TX 77005, USA; 4 Department of Materials Science and NanoEngineering, Rice University, Houston, TX 77005, USA

## Abstract

We have detected trace amounts of molecules of antibiotics (kanamycin sulfate) dispersed on metasurfaces with terahertz (THz) spectroscopy. Utilizing the extraordinary optical transmission resonance of an array of square-shaped slits on a silicon substrate at ~0.3 THz, we were able to monitor varying concentrations of kanamycin sulfate as low as ~100 picogram/L. In contrast, the lowest detectable concentration of kanamycin sulfate on silicon without any metallic structure was ~1 gram/L. This dramatic ~10^10^ times enhancement of sensitivity is due to the near-field enhancement of THz electric fields by the metamaterial structure. This result thus demonstrates the power and usefulness of metamaterial-assisted THz spectroscopy in trace molecular detection for biological and chemical sensing as well as for food product quality and safety inspection and control.

The demand for new chemical and biological sensing methods with higher sensitivities for the effective detection of trace amounts of molecules has been continuously growing for diverse purposes such as food monitoring, environmental science, health care, and national security. In particular, a simple, non-destructive, sensitive, and rapid technique is being sought for detecting antibiotics. While antibiotics are useful for destroying various bacteria and pathogenic microorganisms, the overuse of antibiotics has triggered the development of bacterial resistance, resulting in antibiotic residues in food of animal origin; this is an important health risk^1^, and to improve consumer protection, maximum residue limits and national monitoring plans and protocols have been implemented in different countries. Microbiological assay^2^, chromatography^3^, and capillary electrophoresis^4^ have been used for antibiotics detection because of their excellent sensitivity and selectivity, but they are destructive, and in some cases, require special sample pre-treatment^5^. Other methodologies based on immunology assay, including immunochemistry^6^ and biosensors^7^, are time-saving, sensitive, and highly selective, and can be carried out *in situ*; unfortunately, however, false-positive results hamper these methods from being widely used^8^.

Terahertz (THz) spectroscopy has emerged as a powerful technique for studying diverse materials, including organic- and bio-molecules that resonantly respond to THz radiation^9^. In particular, THz time-domain spectroscopy (THz-TDS) has been shown to have superb signal-to-noise ratios (SNRs), ideal for biosensing and identification^10^, including far-infrared vibrational modes of DNA components^11^, minute changes in biological cell monolayers^10^, and bioaffinity sensing^12^. With the recent wide-spread use of THz-TDS, sensing of chemical and biological materials has become one of the most important THz applications, enabling effective inspection of these substances^12^,^13^,^14^,^15^,^16^,^17^,^18^. Eleven antibiotics that are commonly used in livestock production^19^, as well as three antibiotics and two acaricides (harmful chemical residues in honey), have been detected by THz-TDS with limited sensitivities^20^. While these previous THz studies took advantage of the molecules' THz fingerprints, there are many important antibiotics that do not possess any fingerprints in the THz region.

To enhance THz sensing sensitivities, several approaches have been proposed^21^,^22^,^23^,^24^. One of the most promising routes is to use metamaterials (or their two-dimensional version, metasurfaces). Metamaterials, artificial materials consisting of periodically arranged, sub-wavelength structures, exhibit unique electromagnetic properties that are not available in naturally occurring materials^25^,^26^,^27^,^28^,^29^,^30^,^31^,^32^, which have been utilized in high-sensitivity sensors, ranging from the microwave to the visible range^33^. Recently, metasurfaces operating in the THz frequency range have been used in chemical and biological sensing with enhanced sensitivities to microenvironmental variations, by taking advantage of strongly localized near-fields at resonances^34^,^35^,^36^,^37^,^38^,^39^,^40^,^41^,^42^. On the other hand, selectivity can be implemented with further improved sensitivity by designing the resonance frequency of the metasurface structure to match the “fingerprints” of the sensing target (e.g., some explosives and amino acids)^43^.

In the present work, we have developed a THz metasurface structure that can be utilized for qualitative and quantitative biological sensing. We use a square-shaped metallic extraordinary optical transmission (EOT) structure to enhance THz transmittance, which shows an electromagnetic resonant response at ~0.3 THz. By using this device with THz-TDS, we were able to detect a trace amount of antibiotics, kanamycin sulfate, as small as 100 picogram/liter (100 pg/L), which is about ~10^10^ times enhancement of sensitivity compared to a Si wafer without any metasurface structure.

The metasurface structure we employed in this study consisted of a periodic array of square apertures, as schematically shown in Figure 1a. The device possesses a strong resonance at ~0.3 THz, at which the transmission is ~50% for an aperture array with an open-area fraction of 1.4%. A scanning electron microscopy (SEM) image of the fabricated metallic square aperture arrays with width *w* ~ 1 μm, side length *l* ~ 100 μm, and period *p* ~ 150 μm is also shown in Figure 1b. We performed THz transmission measurements after coating the device with kanamycin sulfate solutions (see Figures 1c and 1d). The solutions were prepared by mixing the kanamycin sulfate with distilled water, and, for each measurement, a volume of ~30 μL was deposited on the metasurface and dried in air.

Because the EOT effect arises from strong local-field concentration due to the metasurface structure, the THz transmission through the structure is highly sensitive to any change around the aperture^42^, such as the presence/absence of kanamycin sulfate molecules. We performed a 3D finite-difference-time-domain simulation using software from Lumerical Solutions, Inc., with a normal-incidence broadband THz wave transmitting through the square gold aperture on an intrinsic silicon substrate. The simulation indeed shows strong electric-field enhancement in and near the aperture (see Supplementary Information), which leads to an ultrahigh THz detection sensitivity, as described below.

Figures 2a and 2b demonstrate the dramatic effect of the metasurface on the detection of kanamycin sulfate through THz transmission spectroscopy. Figure 2a shows transmitted THz amplitude spectra, obtained through Fourier transform of time-domain signals, for a silicon wafer without any metasurface with (red solid line) and without (black dashed line) kanamycin sulfate deposited at a concentration of 0.1 g/L; there is no obvious difference between the two spectra. However, with the incorporation of the EOT structure, deposition of kanamycin sulfate at a miniscule concentration (only 10 ng/L) results in a visible THz amplitude change, as seen in Figure 2b.

THz sensing of kanamycin sulfate was performed through measurements of the transmission change as a function of kanamycin sulfate concentration, ranging from 0 to 10 ng/L (water, 1 pg/L, 10 pg/L, 0.1 ng/L, 1 ng/L and 10 ng/L). Figure 3a shows the fractional transmission change of the metamaterial structure, Δ*T/T*_0_, versus frequency for different concentrations: 1 pg/L (red), 10 pg/L (green), 0.1 ng/L (blue), 1 ng/L (cyan) and 10 ng/L (pink). Here, Δ*T* ≡ |*T*_kana_ − *T*_water_|, where *T*_kana_ and *T*_water_ are intensity transmission spectra for the kanamycin sulfate samples and for the pure water sample, respectively, and *T*_0_ is the peak transmittance for the pure-water deposited sample. Significant peak transmittance changes (up to ~16%) are observed. The transmission spectra of water were undistinguishable from that of bare metasurface with no water. Figure 3b shows that the peak transmittance changes with kanamycin sulfate concentration from *T* ≈ 47% at 1 pg/L to 51% at 10 ng/L. Considering the system stability, combined with principal component analysis (see Supplementary Information), we estimate the minimum detectable kanamycin sulfate concentration to be ~100 pg/L with 99% confidence interval.

For comparison, we also performed similar measurements and data analysis for kanamycin sulfate deposited directly on the silicon wafer without any metamaterial (see, e.g., Figure 2a). We estimate that the minimal detectable concentration in that case is as large as 1 g/L, i.e., ~10^10^ times higher than that for the metasurface case. These results show that the resonant structure presented here is universally applicable to other types of antibiotics because the detection mechanism only relies on the dielectric properties of the antibiotics. To further improve sensitivity, selectivity can be implemented by designing a suitable metasurface once something is known about the response of the sample.

In summary, we developed a novel THz metasurface device based on extraordinary transmission, which can be effectively utilized in biological sensing. As an example, we demonstrated detection of kanamycin sulfate with ultrahigh sensitivity up to 100 pg/L concentration, corresponding to an enhancement factor of ~ 10^10^ compared to detection with a silicon wafer without metasurface. This is potentially applicable for real-time screening procedures suitable for product quality and safety inspection addressing the need for highly sensitive, reliable, and noninvasive sensing techniques to detect chemical and biological objects in real systems with aqueous solutions.

## Methods

### Device Fabrication

The pattern is first defined on the electron-beam (E-beam) resist SU-8 on a 500-μm-thick high resistivity silicon substrate by E-beam lithography. A 100-nm-thick gold film is then evaporated on the patterned resist, followed by a lift-off process^44^.

### Terahertz Time Domain Spectroscopic (THz-TDS) Measurement

The THz-TDS was used to characterize the electromagnetic responses, which is in the transmission geometry. THz pulse is generate by an GaAs wafer excited by a femtosecond Ti:sapphire laser beam. The transmitted THz pulse is detected by a low-temperature grown GaAs photoconductive antenna. The measurements were performed at room temperature in a dry air atmosphere to eliminate the possible disturbance from water (0% humidity). The high-resistivity silicon substrate was used as the reference. The samples coated with kanamycin sulfate and reference substrate were mounted at normal incidence to the THz beam. The samples were rinsed thoroughly using distilled water and dried with nitrogen gas flow before every spectrum collection. After dry, the transmission spectra were measured and compared before and after the coating. Every sample and reference was repeatedly measured five times, according to reference-sample-reference-sample procedure controlled by moving stage. The time domain signals were truncated at 14 ps after the peak of the initial pulse to remove the signal reflected from the back of the substrate to do Fourier transform. The averaged transmission from five times' repeat was used for further analysis.

## Author Contributions

L.X. and W.G. performed the measurements presented in this manuscript. L.X., W.G. and J.K. analyzed the data. J.S. made the metasurface structure. Y.Y. and J.K. provided overall supervision and guidance on the experimental aspects. All authors contributed to data analysis and interpretation as well as the writing of the manuscript.

## Supplementary Material

Supplementary Information Simulation and Data Analysis

## Figures and Tables

**Figure 1:**
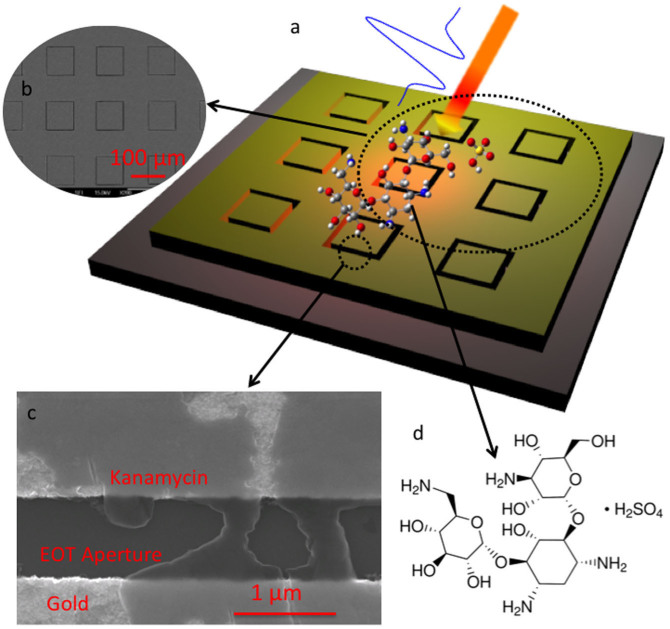
Metasurface-assisted THz sensing of kanamycin sulfate. (a), Schematic of the metasurace structure with kanamycin sulfate molecules deposited on the surface. (b) and (c), SEM images of the metasurface. (d), Kanamycin sulfate molecular structure.

**Figure 2:**
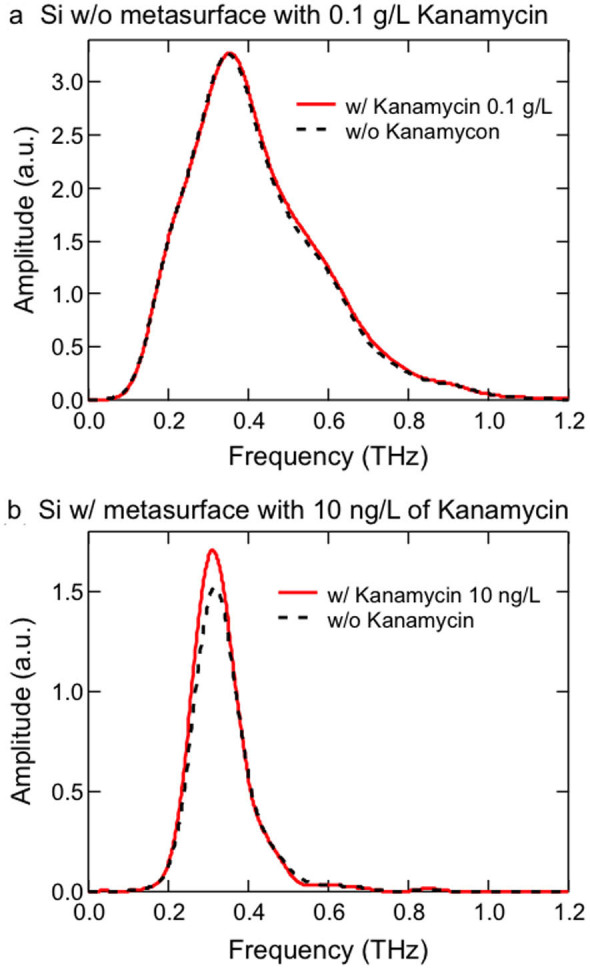
THz amplitude spectra showing the effect of metasurface structure on the detection of kanamycin sulfate. (a), A silicon wafer with no metasurface, without kanamycin sulfate (black dashed curve) and with kanamycin sulfate deposited at a concentration 0.1 g/L (red solid curve), showing no obvious change in transmission. (b), A silicon wafer with metasurface, without kanamycin sulfate (black dashed curve) and with kanamycin sulfate deposited at a very small concentration 10 ng/L (red solid curve), showing a visible enhancement of transmission at the metasurface resonance (at ~0.3 THz).

**Figure 3:**
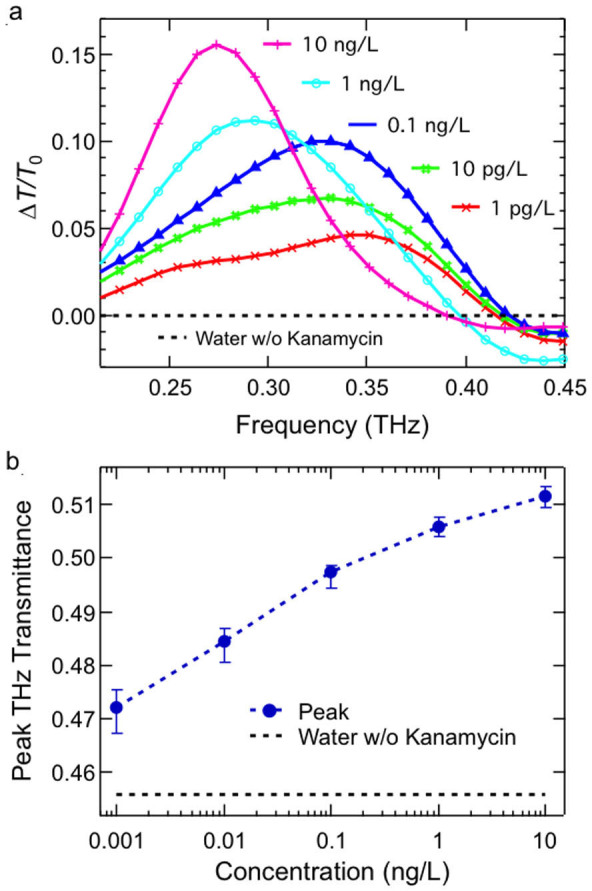
(a), Fractional change of transmittance versus frequency for kanamycin sulfate with 1 pg/L (red), 10 pg/L (green), 0.1 ng/L (blue), 1 ng/L (cyan), and 10 ng/L (pink) concentrations. (b), THz transmission peak value versus concentration.
