# Supplementary material for: Extraordinary sensitivity enhancement by metasurfaces in terahertz detection of antibiotics
Source: Sci Rep. 2015 Mar 2;5:8671. doi: 10.1038/srep08671 (PMC4345331; doi:10.1038/srep08671)
Supplement: Supplementary Information — Simulation and Data Analysis [file srep08671-s1.pdf]

Supplementary Information

Extraordinary sensitivity enhancement by metasurfaces in terahertz detection of antibiotics

Lijuan Xie[1‡], Weilu Gao[2‡], Jie Shu[2], Yibin Ying[1*], and Junichiro Kono[2-4*]

[1]College of Biosystems Engineering and Food Science, Zhejiang University, 866 Yuhangtang Road, Hangzhou 310058, P. R. China, [2]Department of Electrical and Computer Engineering, [3]Department of Physics and Astronomy, and [4]Department of Materials Science and NanoEngineering, Rice University, Houston, TX 77005, USA

‡These authors contributed equally to this work.

**Corresponding author. Yibin Ying, Phone & FAX: 0086-571-88982885, Email: yingyb@zju.edu.cn, Junichiro Kono, Phone: 001-713-3482209, FAX: 001-713-3483091, Email: kono@rice.edu*

1. Numerical Simulation of Metasurface

We performed a 3D finite-difference-time-domain simulation using software from Lumerical Solutions, Inc., with a normal-incidence broadband THz wave transmitting through the square gold aperture on an intrinsic silicon substrate. The distribution of the electric field magnitude is shown in Figures S1a (top view) and S1b (side view). The electric field magnitude is normalized to the maximum value within the metasurface. A typical dipole-like distribution is seen.[1]

2. Principal Component Analysis

To determine the minimum detectable kanamycin sulfate concentration, we employed the method of principal component analysis (PCA)[2]. As a multi-dimensional scaling method, PCA provides a way of visualization of similarities among datasets. Through an orthogonal coordinate transformation, PCA reduces the dimension of datasets to, for example, two, thus projecting the original multidimensional datasets onto a two-dimensional (2D) plane. The original data have the largest variations along the two coordinates after the transformation, so that most information will not be affected by the dimensional reduction. The coordinate values in the new coordinate system are usually referred to as the principle component scores and denoted as PC1 and PC2.

For our THz measurements, each transmittance spectrum was represented as a multidimensional vector, which was then analyzed and illustrated through PC1 and PC2 on a 2D plane[3]. More specifically, a total of 25 raw spectra (five spectra per kanamycin sulfate concentration) were analyzed using the PCA method, and the results are visualized in Figure S2a; PC1 and PC2 here account for 99.94% variations. Similar data will form a cluster in the 2D plot. Especially for the samples with 0.1 ng/L (blue solid squares), 1 ng/L (cyan diagonal crosses), and 10 ng/L (pink open rhombi) concentrations, obvious three well-separated clusters can be observed, without overlapping. From Figure S2a, it can be also seen that the samples with 1 pg/L (red crosses) and 0.01 ng/L (green open triangles) concentrations cannot be distinguished and classified. Therefore, we conclude that the minimum detectable kanamycin sulfate concentration on metasurfaces is 0.1 ng/L.

For comparison, we also performed a similar PCA analysis for the measured transmittance data for kanamycin sulfate directly on the silicon wafer in the same frequency

range as that for metamaterials, as shown in Figure S2b. In this case, two distinguishable clusters are seen. Data points corresponding to concentrations less than 1 g/L are not distinguishable. Therefore, we conclude that the minimum detectable concentration in this case is 1 g/L, which is ~10^{10} times higher than that found in the metasurface case in Figure S2a.

References:

1. Shu, J. *et al*. High-contrast terahertz modulator based on extraordinary transmission through a ring aperture. *Opt. Express* **19(27)**, 26666-26671 (2011).
2. Jolliffe, I. *Principal Component Analysis* (John Wiley & Sons, Ltd, 2005).
3. Park, B. *et al*. Near-infrared diffuse reflectance for quantitative and qualitative measurement of soluble solids and firmness of delicious and Gala apples. *Trans. ASAE* **46(6)**, 1721-1731 (2003).

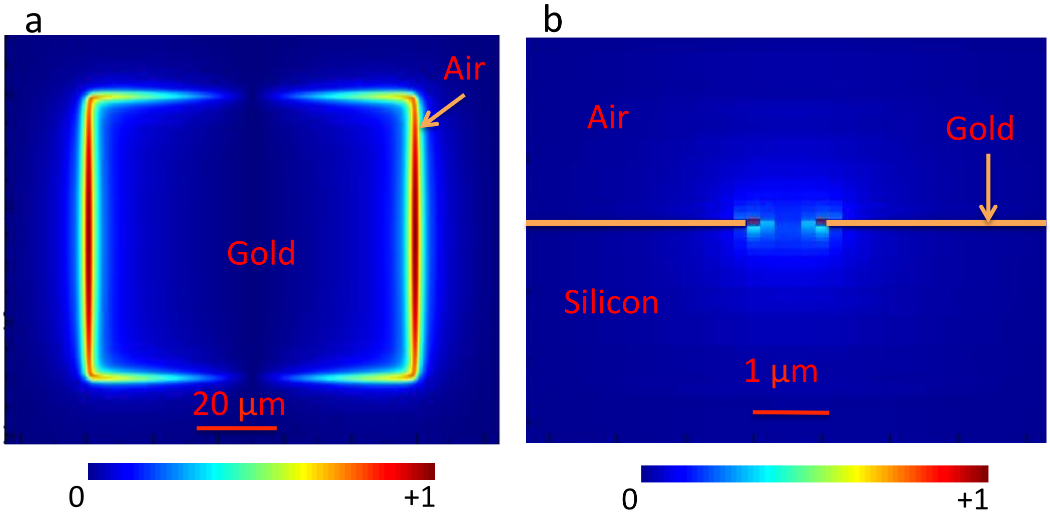

Supplementary Figure 1: The simulated field distribution. a, Top view of one unit of the metasurface structure on a silicon substrate. **b,** Side view of one unit of the metasurface structure on a silicon substrate.

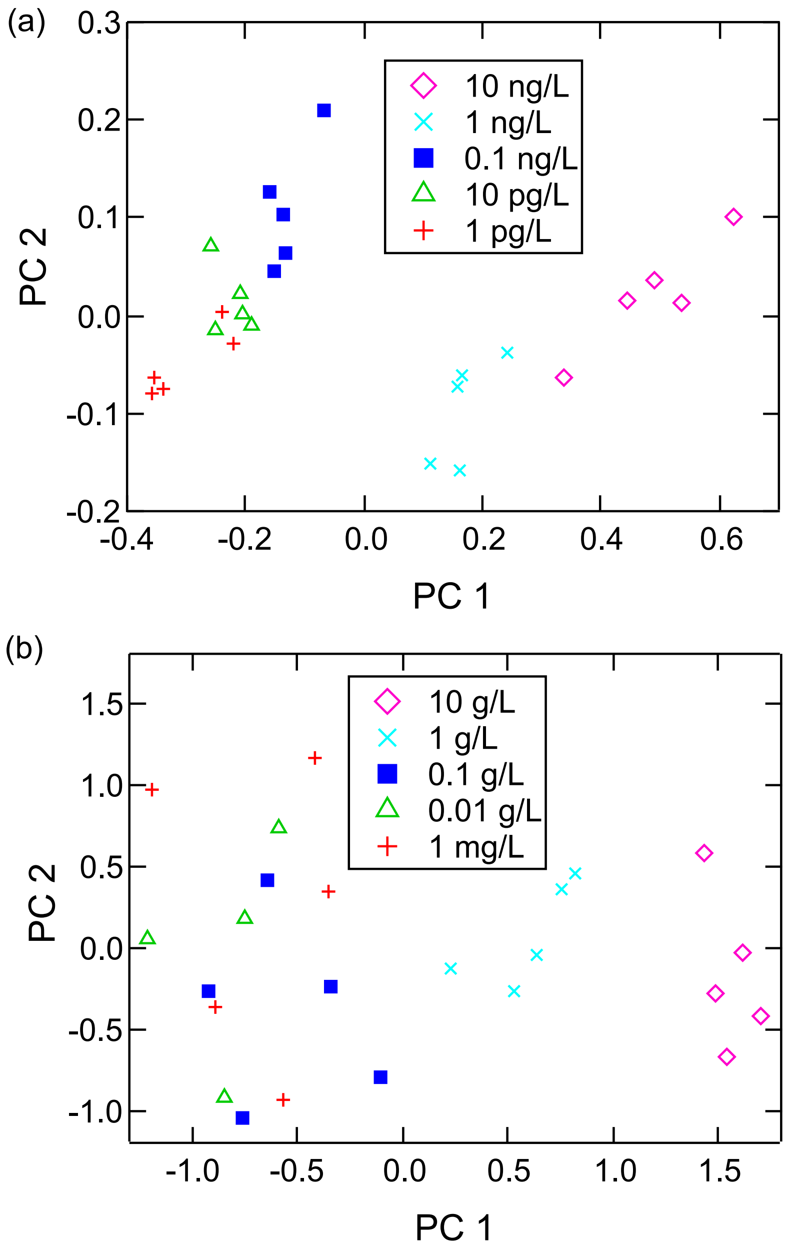

Supplementary Figure 2: a, PCA plot of THz data for kanamycin sulfate on metasurface. Three distinguishable clusters of points are visible, but the lowest two concentration data are not distinguishable, determining the minimum detectable concentration to be 0.1 ng/L. **b**, PCA plot of THz data for kanamycin sulfate on silicon wafer surface. The minimum detectable concentration is 1 g/L.
